# All-Optical Formation and Manipulation of Microbubbles on a Porous Gold Nanofilm

**DOI:** 10.3390/mi11050489

**Published:** 2020-05-10

**Authors:** Qin Cao, Tianli Wu, Xixi Chen, Zhiyong Gong, Ahao Wen

**Affiliations:** Institute of Nanophotonics, Jinan University, Guangzhou 511443, China; caoqin@jnu.edu.cn (Q.C.); wutianli@stu2017.jnu.edu.cn (T.W.); chenxixi@stu2017.jnu.edu.cn (X.C.); zhiyong@stu2018.jnu.edu.cn (Z.G.)

**Keywords:** optical manipulation, microbubbles, plasmonics, gold nanofilm, hot spots

## Abstract

Microbubble generation and manipulation in aqueous environments are techniques that have attracted considerable attention for their microfluidic and biological applications. Ultrasonic and hydrodynamic methods are commonly used to form and manipulate microbubbles, but these methods are limited by the relatively low precision of the microbubble sizes and locations. Here, we report an all-optical method for generation and manipulation of microbubbles with ~100 nm precision by using “hot spots” on a porous gold nanofilm under the illumination of near-infrared focused laser beam. The microbubble diameter ranged from 700 nm to 100 μm, with a standard deviation of 100 nm. The microbubbles were patterned into two-dimensional arrays, with an average location deviation of 90 nm. By moving the laser beam, the microbubbles could be manipulated to a desired region. This work provides a controllable way to form and manipulate microbubbles with ~100 nm precision, which is expected to have applications in optofluidic and plasmonic devices.

## 1. Introduction

Controllable formation and manipulation of microbubbles [1,2,3,4] are crucial techniques for numerous physical and biomedical applications, such as photoacoustic imaging [5,6], cancer surgery [7,8], drug delivery [9], and cell manipulation [10,11]. Ultrasonic and hydrodynamic methods, which rely on the acoustic and shear forces on a liquid–liquid or liquid–air interface, are commonly used to generate and manipulate microbubbles [12,13,14,15]. These methods can produce high-throughput microbubbles with an efficiency of approximately 10^6^ bubbles per second. However, complex external systems, such as ultrasonic generators or flow control devices, are usually needed. Moreover, the size of the microbubbles is limited to several micrometers and the location of the formed microbubbles is usually random. In addition to ultrasonic and hydrodynamic methods, optical methods, such as using optical tweezers, have been also proposed by Prentice [16] and Jones et al. [17]. However, the size and location precision of these methods need to be improved.

Recently, noble metal nanostructures [18,19,20] have emerged as alternative candidates to generate and manipulate microbubbles, which can serve as nanoscale heat sources under the illumination of a pulsed laser [7,21], continuous-wave (CW) laser [22,23], or even sunlight [24] by absorbing photoenergy via plasmon resonance. Differing from floating bubbles generated by ultrasonic and hydrodynamic methods, plasmonic microbubbles are attached to the substrate and possess a truncated spherical shape, making it feasible to change their sizes and locations by remote control [25]. This effect enables the microbubble to act as pump, valve, or lens in optofluidics [26,27,28,29], or to manipulate and fabricate colloidal particles with high mass transfer efficiency [30,31]. However, it is still challenging for noble metal nanostructures to generate and manipulate microbubbles with 100-nm precision of bubble size and location.

**Figure 1:**
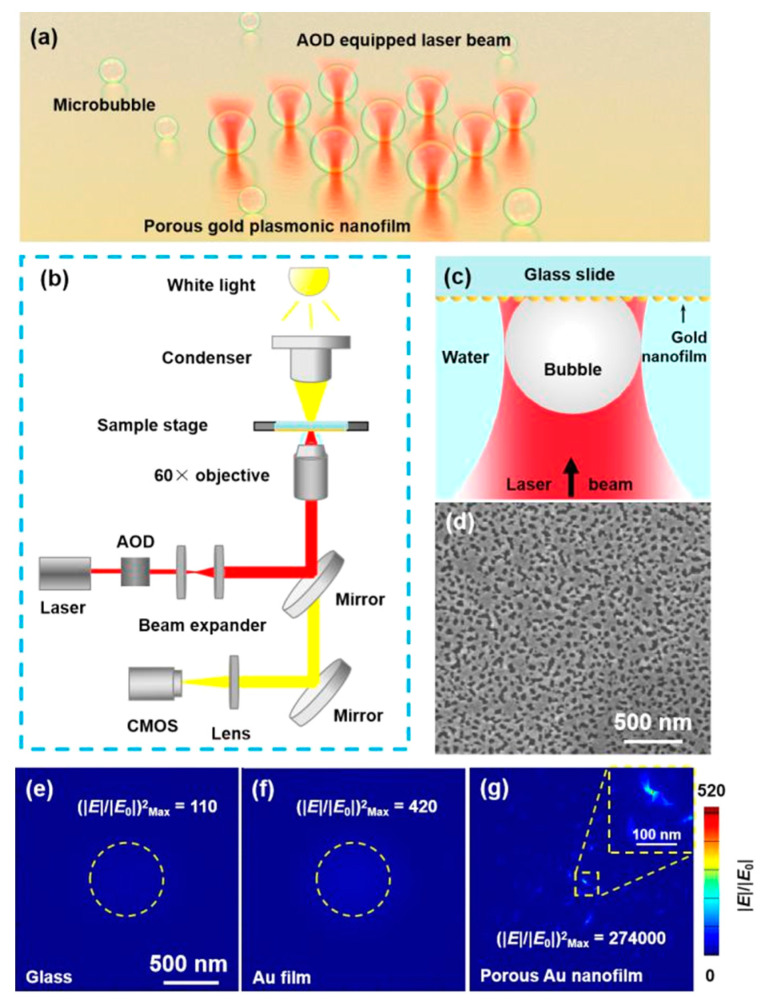
Working mechanism of the formation and manipulation of microbubbles on a porous Au nanofilm. (**a**) Illustration of the optical formation of a microbubble array. (**b**) Schematic of the experimental setup. (**c**) Schematic of bubble generation at the interface between water and the porous Au nanofilm. (**d**) SEM image of the surface geometry of the porous Au nanofilm. Simulated electrical field distributions on (**e**) glass, (**f**) a solid Au film, and (**g**) the porous Au film. The light intensity is in direct proportion to (|E|/|E_0_|)^2^.

In this work, we propose a nanometer-precision method to form and manipulate microbubbles on a porous gold (Au) nanofilm under illumination by a 1064 nm focused laser beam. The optical intensity of the “hot spot” formed by the laser on the porous Au nanofilm is enhanced by 650 times compared with that of a solid Au nanofilm. This high field enhancement allows microbubble size and location precision of 100 and 90 nm, respectively, to be achieved. Arbitrary microbubble patterns composed of multiple microbubbles (as shown in Figure 1a) with well-controlled size and position are generated by changing the laser power and moving the laser beam using an acousto-optic deflector (AOD). Furthermore, optical manipulation and fusion of microbubbles at the interface between the nanofilm and water is achieved by scanning the laser beam.

## 2. Materials and Methods

### 2.1. Sample Preparation

A porous Au nanofilm was deposited directly on a glass slide by conventional ion-beam sputtering as follows. First, a glass slide was cleaned by sonication in ethyl alcohol for 20 min and immersion in ultrapure water three times, and then dried under a flow of N_2_. The clean glass slide was sputtered with a thin layer of Au nanoparticles (NPs) using a sputtering apparatus (ETD-2000C, Elaborate Technology Development, Beijing, China) to obtain an Au NP-coated substrate. During sputtering, the base pressure was kept at 0.1 mbar and the ion current was set at 16 mA. Sputter deposition was conducted for 5 min. The surface morphology of the Au NP-coated substrate was observed by a scanning electron microscope (SEM). The thickness of the sputtered porous Au layer was about 40 nm.

### 2.2. Experimental Setup

The experiments were conducted using an inverted microscope (Eclipse Ti-U, Nikon Corporation, Tokyo, Japan) equipped with an AOD scanning optical tweezer system (Tweez250si, Aresis d.o.o., Ljubljana, Slovenia), as depicted in Figure 1b. A 60× water immersion objective with a numerical aperture of 1.0 was used to focus the irradiation laser and observe the formation process of microbubbles. The sample was inverted and then placed on the stage so that the porous Au nanofilm was in contact with ultrapure water dripped on the objective area. A CW laser with a wavelength of 1064 nm in the optical tweezer system was focused on the sample through the objective to generate heat, and thus produce microbubbles at the interface between water and the Au nanofilm. The spot size of the laser was about 1.3 μm. The spot position and laser power could be arbitrarily changed by the AOD at a maximum frequency of 100 kHz. A white light source with a bright-field condenser illuminated the substrate from the top. The transmission images were captured by a complementary metal-oxide semiconductor camera and visualizations were recorded at 20 frames per second (fps).

### 2.3. Simulation of Electromagnetic Field Enhancement

To demonstrate the electromagnetic field enhancement of the porous Au nanofilm, three-dimensional electromagnetic simulations were conducted by the finite-difference time-domain (FDTD) method with the FDTD Solutions commercial software (Lumerical Solutions Inc., Vancouver, Canada). The geometry of the porous Au nanofilm was imported from the SEM images and the optical constants of Au were taken from the work of Johnson and Christy [32]. The refractive index of water and glass was taken from Palik’s report [33]. A Gaussian beam with a wavelength of 1064 nm was used to illuminate the substrate, which matched the experimental conditions. The override mesh cell was set to 3 × 3 × 3 nm. Contrast simulations were conducted with the beam directly illuminating the glass substrate and solid Au film.

## 3. Results and Discussion

### 3.1. Plasmonic Enhancement of the Porous Au Nanofilm

As depicted in Figure 1c, a single microbubble could be generated at the interface of water and the porous Au nanofilm when the CW laser beam was focused on the substrate, due to the optical absorption and the heat from photothermal effect. The amount of the generated heat was basically determined by the optical absorption. To show the plasmonic enhancement of the porous Au nanofilm, FDTD simulations of three models were conducted. The configuration of the porous Au nanofilm was constructed from the imported SEM image shown in Figure 1d. The lighter part on the SEM image was covered by gold nanoparticles and the dark part was the substrate. The coverage rate of gold nanoparticles was measured by Image J (National Institutes of Health, Bethesda, Rockville, MD, USA), which was approximately 71.76%. Figure 1e–g display top views of the electrical field distributions of the glass substrate, solid Au film, and porous Au nanofilm, respectively. The electrical field was strongest at the center of focus on the plane ~200 nm above the glass surface and solid Au nanofilm, and the amplitude of the electric field was enhanced by 10.7 and 20.5 times, respectively. By comparison, the plasmonic enhancement on the porous Au nanofilm induced by the network of hot spots was clearly visible and the strongest enhancement of 523.8 times was located at the resonance point near the center of the surface. Because the light intensity was in direct proportion to (|E|/|E_0_|)^2^, the enhancement of light intensity on the porous Au nanofilm was about 2500 and 650 times that of the glass substrate and solid Au nanofilm, respectively. This was beneficial for generating heat on the nanostructure. Additionally, the enhancement at the outer ring on the porous Au nanofilm decreased significantly, so that heat was mainly generated at the central position, which was helpful for improving the size and location precision of induced microbubbles [34].

### 3.2. Dynamic Process of Microbubble Formation and Shrinkage

Under CW laser illumination, the temperature of water near the plasmonic NPs rapidly rose to more than 220 °C within a few nanoseconds to microseconds [23]. Because the temperature was much higher than the boiling point of water at 1 atm, the surrounding water evaporated and generated a bubble through heat transfer, which is known as superheating. The thermal conduction from the heating source, i.e., the porous Au nanofilm, to the microbubble generated in situ and surrounding liquid has been discussed extensively [35,36]. According to Fourier’s law:

(1) $$\frac{dQ}{dt}=-4\pi kR^{2}\partial_{r}T$$

where *Q* is the generated heat power (J), *k* is the thermal conductivity of the surrounding liquid (J·s^−1^·m^−1^·K^−1^), *R* is radius of the bubble, and $\partial_{r}T$ is the temperature gradient at the water–Au interface. Assuming that $\partial_{r}T\approx \Delta T/R$, it gives:

(2) $$\frac{dQ}{dt}\approx -4\pi kR\Delta T$$

where Δ*T* is the temperature difference (K) between the bubble and the fluid far from the bubble, which is approximately the same under identical conditions. Therefore, the heat transfer from the bubble is proportional to *R*.

**Figure 2:**
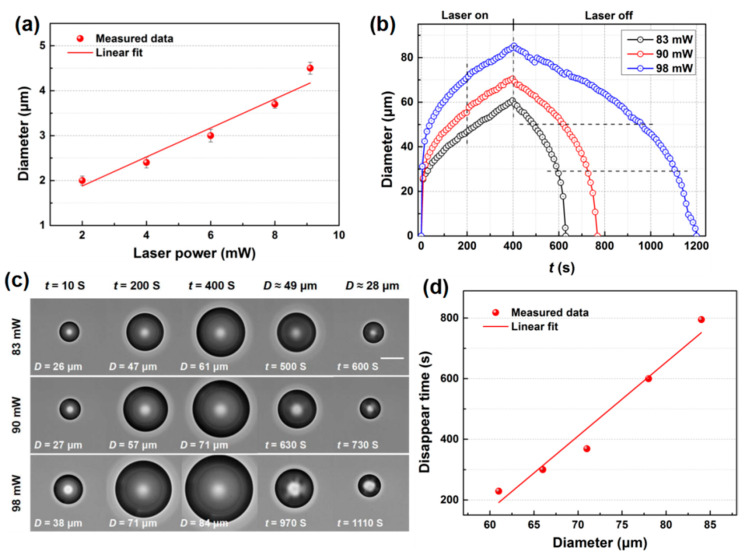
(**a**) The dependent relationship of the microbubble diameter with the laser power. (**b**) Evolution of expanding bubbles at laser powers of 83, 90, and 98 mW (**c**). Frames showing expanding and collapsing velocities and microbubble diameter induced by different laser powers. Scale bar: 30 μm (**d**). The dependent relationship of the time it took for a microbubble to disappear on the microbubble size (*n* = 3).

In the bubble generation experiments, we observed that a stable microbubble formed instantly when the laser illuminated the substrate surface and when the laser was turned off the microbubble instantly disappeared, which indicated that it was formed by water vapor [31] with relatively low laser power. The size of the microbubbles in the steady state increased with the laser power because more water vapor was generated. A linear dependence relationship could be summarized between the size of the stable microbubbles and the laser power, as plotted in Figure 2a. The results were the averages of three measurements.

When the laser power was further increased, expanding microbubbles were observed because of superheating [37]. Figure 2b shows the diameter (*D*) of the expanding microbubbles as a function of time (*t*) under CW laser illumination at different power intensities (also see Supplementary Video S1). *D* was measured from the visualization frames. Some of these frames of typical moments during the process are presented in Figure 2c. The *D* of the microbubbles kept growing under laser illumination at 83, 90, and 98 mW, and then gradually shrank once the laser was turned off. Additionally, the higher the laser power, the more quickly the microbubble grew, and the bigger the microbubble was, the more time it took to collapse. In the first 10 s, the *D* of the microbubbles generated at all three laser powers increased to 26, 27, and 38 μm at velocities of 2.6, 2.7, and 3.8 μm·s^−1^, respectively. Then, the bubble expansion slowed down. At 400 s, the diameters of the microbubbles were 61, 71, and 84 μm, with average increasing velocities of 0.1525, 0.1775, and 0.21 μm·s^−1^, respectively. The microbubbles kept expanding because the focus of the laser beam was simultaneously moved to the maximum *D* of the bubble, resulting in larger areas of NPs being illuminated and more heat being generated. When the laser was turned off, the microbubbles generated at powers of 83, 90, and 98 mW took 229, 369, and 795 s to collapse, respectively. It would take more time for bigger microbubbles to disappear. The relationship between the time it took for a microbubble to disappear and the microbubble size is plotted in Figure 2d. The lifetimes of more than several minutes demonstrated that these microbubbles were composed of not only water vapor but also air dissolved in the water. Bubble expansion in the early explosive stage was dominated by water vapor, whereas in the latter stage this was induced by the dissolved air from the surrounding water. When the heating source was turned off, the air molecules gradually transferred into the liquid until the bubble completely disappeared. This behavior was in accordance with that observed in a former study [38].

### 3.3. Formation of Microbubble Patterns with Multiple Microbubbles

To form microbubble patterns composed of multiple microbubbles with high precision, the location of the laser spot was modulated by the AOD, and each site received a time slot of illumination with the duration being inversely proportional to the number of sites. When the shifting frequency (*f*_s_) was set and the laser spot kept shifting between sites and travelling along the loop, each site was illuminated for 1/*f*_s_ in one circulation. The second illumination at a site only reached after the laser spot traversed the rest of the loop for (*n*−1)/*f*_s_; that is, the illumination rate of a certain site is *f*_s_/*n*, where *n* is the number of sites. In other words, the laser illumination at a certain site was pulsed. Because the porous Au nanofilm was relatively uniform on the glass substrate and its “hot spots” were well distributed (Figure 1d), any illuminated site generated a microbubble of a corresponding size. Stationary microbubble patterns composed of multiple microbubbles could be formed if the lifetime of a located microbubble was longer than the interval time of the presence and absence of laser illumination.

**Figure 3:**
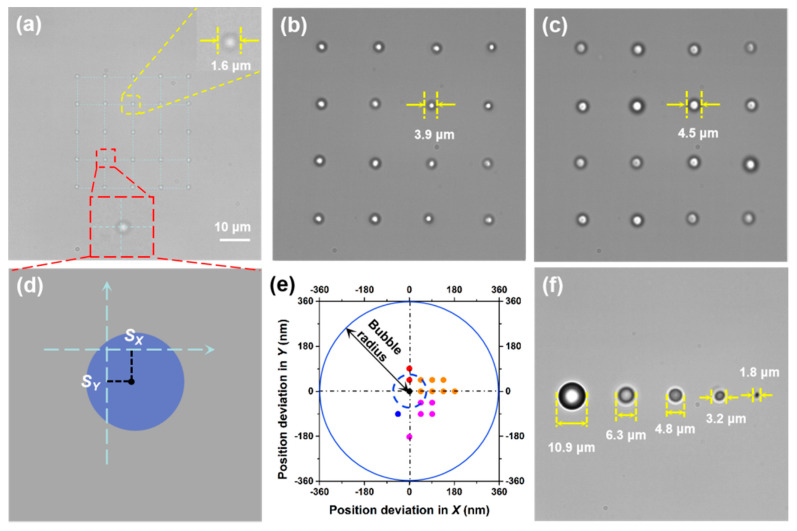
Formation of microbubble arrays. (**a**) A 5 × 5 array of microbubbles with an average diameter of 1.64 μm. (**b**) A 4 × 4 array of microbubbles with an average diameter of 3.88 μm. (**c**) A 4 × 4 array of microbubbles with an average diameter of 4.52 μm. (**d**) Illustration of the position deviation in X (*S*_X_) and Y (*S*_Y_) directions for an individual microbubble. (**e**) Position deviations in X and Y directions for the microbubbles in the 2D array; (**a**) the dashed blue circle indicates the average deviation of ~90 nm from the target positions. (**f**) A row of microbubbles of different sizes (*n* = 3).

Figure 3a shows a 5 × 5 array of microbubbles with a diameter of 1.64 ± 0.10 μm produced by a shifting laser spot with a power of 2 mW. A 4 × 4 array of microbubbles with *D* of 3.88 ± 0.12 μm generated by a shifting laser spot with a power of 38 mW is presented in Figure 3b. In addition, Figure 3c depicts a 4 × 4 array of microbubbles with *D* of 4.52 ± 0.21 μm formed by a shifting laser spot with a power of 45 mW. The size accuracy of the microbubbles in the 5 × 5 array was determined by the standard deviation of individual microbubbles from the average value, which was 100 nm. The position accuracy for a single microbubble was estimated by the distance of its center from the target position (Δ*S*), as illustrated in Figure 3d. Figure 3e depicts the deviation of all 25 microbubbles in the 5 × 5 array (three microbubbles were located at the center and several microbubbles deviated by the same distance). All the microbubbles were located very close to their target position, with an average Δ*S* of ~90 nm, i.e., the mean deviation was ~90 nm, which was much smaller than the diameter of the microbubbles. Independent control over the sizes of several microbubbles was also achieved. Figure 3f depicts a row of stable microbubbles with diameters of 10.9, 6.3, 4.8, 3.2, and 1.8 μm, generated when the laser power at each corresponding site was set at 90, 80, 70, 30, and 15 mW, respectively. The shifting frequency of the laser beam in the above examples was set at 100 kHz. In Figure 3a, each microbubble site was illuminated for 10 μs at an interval of 240 μs, and in Figure 3f each microbubble site was illuminated for 10 μs at an interval of 40 μs.

**Figure 4:**
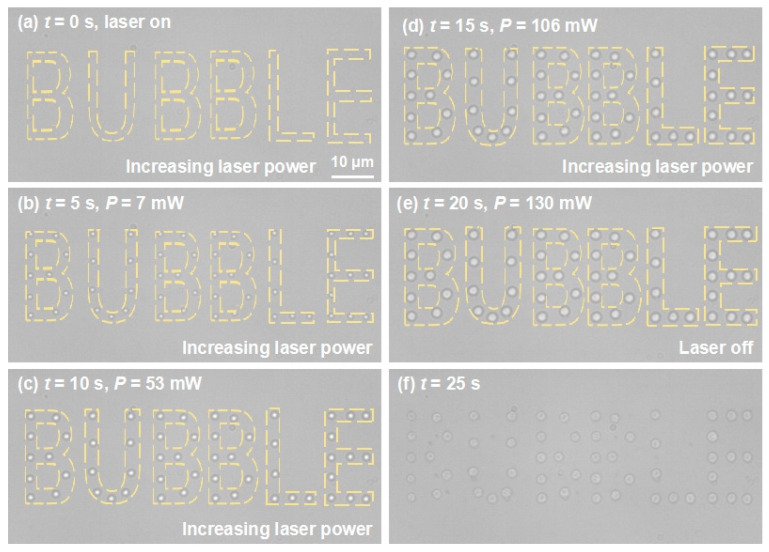
Evolution of the grown microbubble pattern “bubble” with increasing power (*P*) of the laser beam. (**a**) At time *t* = 0 s, the laser was turned on and some microbubbles with *D* of ~700 nm appeared at designed positions. (**b**) At *t* = 5 s, *P* increased to 7 mW and the microbubble diameter in (**a**) grew to 1.4 μm. (**c**) At *t* = 10 s, *P* increased to 53 mW and the *D* of the microbubbles grew to 1.9 μm. (**d**) At *t* = 15 s, *P* increased to 106 mW and the microbubble diameter grew to 2.5 μm. (**e**) At *t* = 20 s, *P* increased to 130 mW, the microbubble diameter grew to 2.6 μm, and then the laser was turned off. (**f**) At *t* = 25 s, the microbubbles collapsed and left traces of fragments from the Au NPs (*n* = 3).

Based on the above findings showing that the microbubble size increased with the laser power and that arbitrary microbubble patterns can be generated with the aid of the AOD, coordinate positions of the word “bubble” were designed on the manipulation area, then the laser was turned on and shifted to each position, travelling in loops. As illustrated in Figure 4a–e, a stationary pattern of microbubbles forming the word “bubble” was generated. As we gradually increased the laser power, the sizes of the microbubbles increased simultaneously (also see Supplementary Video S2). Upon turning on the laser (*t* = 0 s), some microbubbles with *D* of ~700 nm appeared (Figure 4a). When the laser power was increased to 7 mW, a distinct pattern of microbubbles with *D* of 1.4 μm was observed (Figure 4b). When the laser power was increased to 130 mW, *D* of a single microbubble reached 2.6 μm (Figure 4e). Larger microbubbles were obtained as the laser power continued to increase. When the laser was turned off, the microbubbles collapsed and left some traces (Figure 4f), which may be fragments originating from the evaporation of Au NPs, caused by the dramatic temperature increase when the microbubble was formed and the NPs were isolated from the surrounding water and restricted to transfer heat to vapor rather than liquid water [39]. The fragments of AuNPs could act as subtle heat sources to keep the microbubble stable. The above processes confirmed that it was possible to realize arbitrary patterning of microbubbles with highly controlled sizes and positions over a large area.

In this case, 56 microbubbles were simultaneously formed and manipulated. By shifting the laser beam between more locations, more microbubbles could be formed simultaneously. The production rate of this method is dependent on the shifting frequency (at a maximum of 100 kHz) of the laser beam in the optical tweezers system, and the lifetime of single bubble. The laser beam could travel around 10^5^ locations per second at most. For microbubbles that were composed of water vapor and gas, their lifetime could last for several minutes. The bubbles would not collapse before the laser beam travelled a loop of 10^5^ locations in one second. Therefore, the production rate might be estimated to reach 10^5^ per second at most by the theoretical analysis. Although the throughput of this method may not be up to 10^6^ bubbles per second, as stated by ultrasonic and hydrodynamic methods, this is sufficient in most situations for main applications. However, the size of the microbubbles produced in this way is uniform rather than random, as compared with traditional methods. The size limit is reduced from several micrometers to 700 nm, and the position accuracy is a novel concept. The dynamic control of microbubbles at designated positions, with diameters measuring within several micrometers, is beneficial for microfluidic devices; for example, they could act as pumps and valves in microfluidics, especially in narrow environments.

### 3.4. Optical Movement and Fusion of Microbubbles

**Figure 5:**
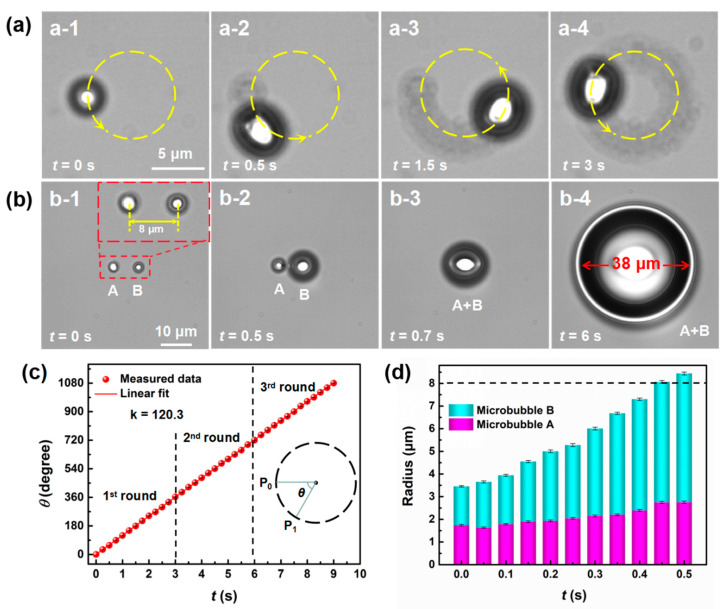
Optical manipulation and fusion of microbubbles. (**a**) Circular movement of a microbubble around a circle with a diameter of 10 μm. (**b**) Fusion process of two adjacent microbubbles. (**c**) Dependence of the travelled angle of the moving microbubble on time. (**d**) Changes of the radius of microbubble A and B during the fusion process.

Movement of microbubbles can be recorded by using either a low shifting frequency or high frame rate. At a fixed frame rate of 20 fps, the shifting frequency was set at 1.8 kHz and the laser spot travelled for loops of 5000 positions along a circular profile with a diameter of 10 μm. The shift (*s*) of the laser spot in one loop was approximately 31.4 μm, so the step of the shift was *s*/5000 = 6.28 nm. As shown in Figure 5a, a microbubble with *D* of 4.5 μm formed at the starting position of the loop under CW laser illumination at a power of 9.1 mW for 0.56 ms. Then, the microbubble appeared to move anticlockwise to the next position along the circle and its shape became elongated (also see Supplementary Video S3). This phenomenon occurred because the initial microbubble collapsed when the laser spot moved away and then a new microbubble formed when the laser spot moved to the next position, so it appeared that the microbubble was moving. Note that it was not a single microbubble that was manipulated to move, but a phenomenon of continuous generation of microbubbles at the leading edge and the collapsing of the previous bubble. The elongation of the microbubble was caused by the fusion of the former collapsing microbubble and the stable microbubble formed under the laser spot at that moment. When two microbubbles were close to each other, they fused into a larger one. As illustrated in Figure 5b, microbubble A with *D* of 3.7 μm formed first by laser irradiation at a power of 8 mW. Microbubble B was formed later by slowly increasing the laser power. When the power of the laser illuminated on microbubble B increased to the same level as that of microbubble A, microbubble B expanded by absorbing vapor from microbubble A, and the two microbubbles fused into one microbubble with an elliptical shape, which then kept expanding (also see Supplementary Video S4). Once the laser stopped, the microbubble collapsed. The angular velocity of the moving microbubble was constant (120.3°·s^−1^) during three loops, as indicated by the slope of the linear fitted curve between the travelled angle and time in Figure 5c. Figure 5d presents the recorded changes of the bubble radius during the fusion process. The radius of microbubble B increased constantly, and when the sum of the radii of the two microbubbles reached 8 μm, the two microbubbles fused gradually. The movement of microbubbles along a designed pathway and the fusion of microbubbles are advantageous actions for manipulation of particles or cells in microfluidics [40,41,42].

## 4. Conclusions

Microbubbles with *D* from 700 nm to 100 μm were formed with nanometer-precision sizing and manipulated with nanometer-precision positioning. The dynamics of a single microbubble at the interface between water and a porous Au nanofilm under the illumination of a 1064 nm CW laser were evaluated by numerical simulation and experimental observations. Microbubble patterns composed of multiple microbubbles were then formed by shifting the laser beam at high frequencies with an AOD. The sizes and positions of the microbubbles in patterns could be independently controlled, with standard deviations of 100 and 90 nm, respectively. Microbubble movement and fusion were also achieved by tuning the shifting frequency and power of the laser beam. The developed technique is attractive for controlled generation and manipulation of microbubbles, and may have practical applications in microfluidic fields.

## Supplementary Materials

The following are available online at https://www.mdpi.com/2072-666X/11/5/489/s1: Video S1: Expansion and collapse of a microbubble. Video S2: Growing the “bubble” microbubble pattern. Video S3: Circular movement of a microbubble. Video S4: Fusion of two adjacent microbubbles.
